# Mapping Sub-surface Distribution of Soil Organic Carbon Stocks in South Africa’s Arid and Semi-Arid Landscapes: Implications for Land Management and Climate Change Mitigation

**DOI:** 10.1016/j.geodrs.2024.e00817

**Published:** 2024-06

**Authors:** Omosalewa Odebiri, Onisimo Mutanga, John Odindi, Rob Slotow, Paramu Mafongoya, Romano Lottering, Rowan Naicker, Trylee Nyasha Matongera, Mthembeni Mngadi

**Affiliations:** 1 School of Agricultural, Earth and Environmental Sciences, Discipline of Geography, University of KwaZulu-Natal, Pietermaritzburg, South Africa; 2 Centre for Integrative Ecology, School of Life and Environmental Sciences, Deakin University, Melbourne, VIC 3125, Australia; 3 Centre for Transformative Agriculture and Food Systems, University of KwaZulu-Natal, Pietermaritzburg, South Africa; 4 Oppenheimer Fellow in Functional Biodiversity, Centre for Functional Biodiversity, School of Life Sciences, University of KwaZulu-Natal, Pietermaritzburg, South Africa; 5 Department of Genetics, Evolution and Environment, University College London, London, UK; 6 Agronomy and Rural Development, School of Agricultural, Earth and Environmental Sciences, University of KwaZulu-Natal, Scottsville, Pietermaritzburg, South Africa

**Keywords:** Soil organic carbon, arid and semi-arid regions, climate action, machine learning, soil management

## Abstract

Soil organic carbon (SOC) stocks are critical for land management strategies and climate change mitigation. However, understanding SOC distribution in South Africa’s arid and semi-arid regions remains a challenge due to data limitations, and the complex spatial and sub-surface variability in SOC stocks driven by desertification and land degradation. Thus, to support soil and land-use management practices as well as advance climate change mitigation efforts, there is an urgent need to provide more precise SOC stock estimates within South Africa’s arid and semi-arid regions. Hence, this study adopted remote-sensing approaches to determine the spatial sub-surface distribution of SOC stocks and the influence of environmental co-variates at four soil depths (i.e., 0-30 cm, 30-60 cm, 60-100 cm, and 100-200 cm). Using two regression-based algorithms, i.e., Extreme Gradient Boosting (XGBoost) and Random Forest (RF), the study found the former (RMSE values ranging from 7.12 t/ha to 29.55 t/ha) to be a superior predictor of SOC in comparison to the latter (RMSE values ranging from 7.36 t/ha to 31.10 t/ha). Nonetheless, both models achieved satisfactory accuracy (R^2^ ≥ 0.52) for regional-scale SOC predictions at the studied soil depths. Thereafter, using a variable importance analysis, the study demonstrated the influence of climatic variables like rainfall and temperature on SOC stocks at different depths. Furthermore, the study revealed significant spatial variability in SOC stocks, and an increase in SOC stocks with soil depth. Overall, these findings enhance the understanding of SOC dynamics in South Africa’s arid and semi-arid landscapes and emphasizes the importance of considering site specific topo-climatic characteristics for sustainable land management and climate change mitigation. Furthermore, the study offers valuable insights into sub-surface SOC distribution, crucial for informing carbon sequestration strategies, guiding land management practices, and informing environmental policies within arid and semi-arid environments.

## 1 Introduction

The increasing prevalence of extreme weather events has heightened global concerns on the existential threat of climate change (Kumar et al., 2021, Jackson, 2023). Consequently, it has now become imperative to implement strategic measures to slow the progression of climate change, mitigate its impacts, and strive to achieve the 1.5 ºC target threshold suggested by the Intergovernmental Panel on Climate Change (Mariappan et al., 2023; Odebiri et al., 2023a; Wang et al., 2021). In this regard, enhancing carbon sequestration capacity and storage has become one of the most effective strategies in climate change mitigation (Lal, 2015). Soil organic carbon (SOC) is one of the largest terrestrial carbon reservoirs (Li et al., 2021; Sahoo et al., 2019), with the capacity to offset between 5 and 20% of all global greenhouse gas emissions (Venter et al., 2021; Odebiri et al., 2020a; Allen et al., 2019; Kenye et al., 2019). Furthermore, SOC plays a fundamental role in preserving soil health, facilitating nutrient cycling, and promoting enhanced plant productivity (Odebiri et al., 2020b; Palm et al., 2014; Doran, 2002). Therefore, disturbances in SOC stocks can have profound and far-reaching bio-physical and environmental consequences, impacting not only the intricate dynamics of the global carbon cycle, but also influencing the soil’s physical, chemical, and biological characteristics (Lamichhane et al., 2019; Sainepo et al., 2018). As a result, the preservation and enhancement of SOC stock has evolved beyond being just a key consideration within ecosystem management and carbon accounting strategies to represent a critical cornerstone within broader global climate change mitigation frameworks (Li et al., 2021; Sahoo et al., 2019).

Appropriate climate and land-use decision-making requires the availability of precise and reliable SOC information (Lorenz and Lal, 2005, Poeplau et al., 2011). Such precise and reliable data provide essential insights into a landscape’s productivity and carbon assimilation potential, while serving as a critical indicator of possible landscape degradation (Chappell et al., 2019, Trivedi et al., 2018). Specifically, SOC provides essential information on soil fertility, nutrient availability, soil structure, moisture retention, and microbial activity (Hou et al., 2020). Collectively, these factors can provide a broad overview of landscape conditions (Hou et al., 2020, Billings et al., 2021). For instance, elevated SOC levels signify healthier soils — which are often characterized by higher rates of nutrient cycling and enhanced water-holding capacity (HUSSAIN et al., 2023). These conditions promote increased vegetation diversity, improved ecosystem resilience, and reduced vulnerability to drought or extreme weather conditions (Lal, 2016). Furthermore, SOC information acts as a key indicator of landscape degradation, as declining levels of SOC can often indicate instances of potential soil erosion, ecosystem degradation, or a loss of soil fertility (Chappell et al., 2019). Thus, closely monitoring fluctuations in SOC becomes imperative for not only preserving landscape health and productivity but also for the maintenance and enhancement of an ecosystem’s carbon assimilation capacity. Nonetheless, due to the complex sub-surface dynamics and uneven distribution of SOC within the soil profile (Jandl et al., 2014), SOC information needs to be adequately captured at multiple soil depths. This is because the soil profile is categorized into three specific soil layers, which include the topsoil (0-10 cm), the subsoil (10-30 cm), and the substratum (>30 cm). Literature has shown a significant variation within SOC within the soil profile, with complex processes affecting its distribution (De Caires et al., 2024, Sarkodie et al., 2023, Rial et al., 2017). Therefore, ensuring informed ecological and climate related decision-making among key stakeholders is often hinged upon the near real-time availability of accurate and reliable SOC information at multiple sub-surface levels.

However, comprehensive SOC data is often limited in arid and semi-arid landscapes, as these areas are often neglected within soil studies, despite their considerable potential for carbon sequestration (Mousavi et al., 2022; Biederman et al, 2017). Encompassing approximately 40% of the world’s terrestrial surface, semi-arid and arid lands play a fundamental role in global carbon cycling (Smith et al., 2019; Schimel, 2010). Although these regions are generally characterized by low rainfall, high temperatures, and sparse vegetation, they contain substantial amounts of SOC (Fensholt et al., 2012). This carbon is primarily derived from the decomposition of available plant litter, root exudates, and other organic matter inputs, which is then stabilized through physical and chemical protection (Chenchouni and Neffar, 2022; Wang et al., 2015). Moreover, these regions have been identified as substantial reservoirs for carbon storage (Mureva et al., 2018). Several studies, for instance, have documented their potential to retain up to 30% of the world’s SOC over extended periods, with SOC predicted to persist at different levels within the soil profile for centuries or millennia (Mureva et al., 2018; Zhang et al., 2013). Additionally, arid and semi-arid environments are often exposed to fluctuations in anthropogenic and environmental processes, which influence the complex SOC dynamics experienced within the different sub-soil levels (Mandal et al., 2020, Ibrahim et al., 2019, Venter et al., 2021). Therefore, it is crucial to acquire a holistic understanding of the underlying processes that govern SOC dynamics within the sub-surface of semi-arid and arid landscapes. This understanding serves to not only evaluate the potential of these regions in climate change mitigation, but also provide a broad indicator of ecosystem health and functioning with the landscapes.

In South Africa, arid and semi-arid regions cover approximately 60% of the country (Mucina and Rutherford, 2006). These regions are crucial to the country’s agricultural sector, as they support livestock grazing and farming of staple crops such as maize and wheat (Jat et al., 2012, McCown et al., 1979). Generally, these regions are predominately characterized by varying and unpredictable rainfall, high temperatures and evapotranspiration rates, leading to high rates of SOC variability throughout the landscape (Fouché, 2017, Mucina and Rutherford, 2006). Recent studies in South Africa (e.g., Dai et al., 2022; Venter et al., 2022; Odebiri et al., 2022a; Odebiri et al., 2023a, Odebiri et al., 2023b) have also noted significant disparities in SOC stocks within the country’s arid and semi-arid landscapes. Although of the high variability has been largely attributed to several environmental factors, that include climate, vegetation cover, topography, and soil properties (Dai et al., 2022; Venter et al., 2022; Odebiri et al., 2022a), literature on sub-surface soil-based information in these regions remains largely unavailable, with poor understanding of the spatial distribution of SOC at different soil depths within landscapes. Existing studies (e.g., Venter et al., 2022; Odebiri et al., 2022a; Odebiri et al., 2023a, Odebiri et al., 2023b) have predominately focused on surface SOC (0-30cm) estimates, neglecting the impact of environmental factors on SOC stocks at lower depths (i.e., 30–200 cm) in these regions.

Subsoil horizons (30–200 cm) contribute more than half of the total global soil carbon stocks (Rumpel et al., 2012; Ngo et al., 2013). Therefore, it is crucial to generate a comprehensive understanding of subsoil carbon dynamics, which can then be integrated into regional carbon estimates (Wang et al., 2016). This is particularly vital due to the lack of consensus surrounding the dynamics of SOC storage and of the relative influence of environmental factors on SOC variability at lower soil depths (Lorenz and Lal, 2014; Scharlemann et al., 2014). Moreover, considering the vulnerability of arid and semi-arid landscapes to increased desertification and soil degradation, there is significant uncertainty on the influence of key SOC storage drivers at various depths (Odebiri et al., 2020). Thus, it is imperative to investigate SOC dynamics and the impact of various environmental variables on SOC distribution at different depths in these environments. Such investigations will offer valuable insights into SOC behaviour at varying soil profiles, leading to improved landscape management and SOC storage strategies and reporting.

In recent years, the prediction of SOC distribution using Digital Soil Mapping (DSM) has become appealing (Ibrahim et al., 2019, Sarkodie et al., 2023, Zhang et al., 2024, Odebiri et al., 2020). This approach uses environmental covariates in concert with predictive models to delineate SOC spatial distribution (Kaya et al., 2022, Odebiri et al., 2023a, Odebiri et al., 2023b). Typically, this framework integrates widely utilized covariates such as digital elevation models (DEMs), climatic variables and remotely sensed (RS) data (Padarian et al., 2019; Lamichhane et al., 2019). The utilization of machine learning (ML) algorithms to explore these covariates have become increasingly popular for SOC prediction (Kaya et al. 2022; Mousavi et al., 2022; Yuan et al., 2020; Ma et al., 2019). ML algorithms, such as decision tree-based models and neural networks are preferred due to their ability to capture nonlinear relationships between SOC and its associated covariates (Singh and Kasana, 2019; Minh et al., 2018). Furthermore, considering that soil properties, including SOC, vary with depth, it is crucial to understand how the performance of ML algorithms and the effect of environmental covariates are affected by depth within arid and semi-arid regions (Minasny et al., 2016; Benke et al., 2020). Hence, this study sought to predict the spatial and sub-surface distribution of SOC stocks in South Africa’s arid and semi-arid lands at four depths (0-30 cm, 30-60 cm, 60-100 cm, and 100-200 cm) and assess the influence of environmental covariates on these using a remote sensing approach. We hypothesized that topo-climatic variables will exert varying influences on the spatial distribution of SOC stocks across different soil depths. Moreover, the performance of two prominent ML algorithms, namely random forest (RF) and extreme gradient boosting (XGBoost), in mapping SOC’s spatial distribution at different soil depths were evaluated. Both RF and XGBoost have demonstrated good performance in predicting the spatial distribution of SOC stocks with acceptable accuracies (Chen et al., 2023; Wiesmeier et al., 2011). These models excel at managing soil datasets of various sizes, addressing multicollinearity, handling missing values, and effectively identifying non-linear relationships between SOC stocks and other predictors (Odebiri et al., 2022a).

## 2 Methodology

### 2.1 The study site

**Figure 1:**
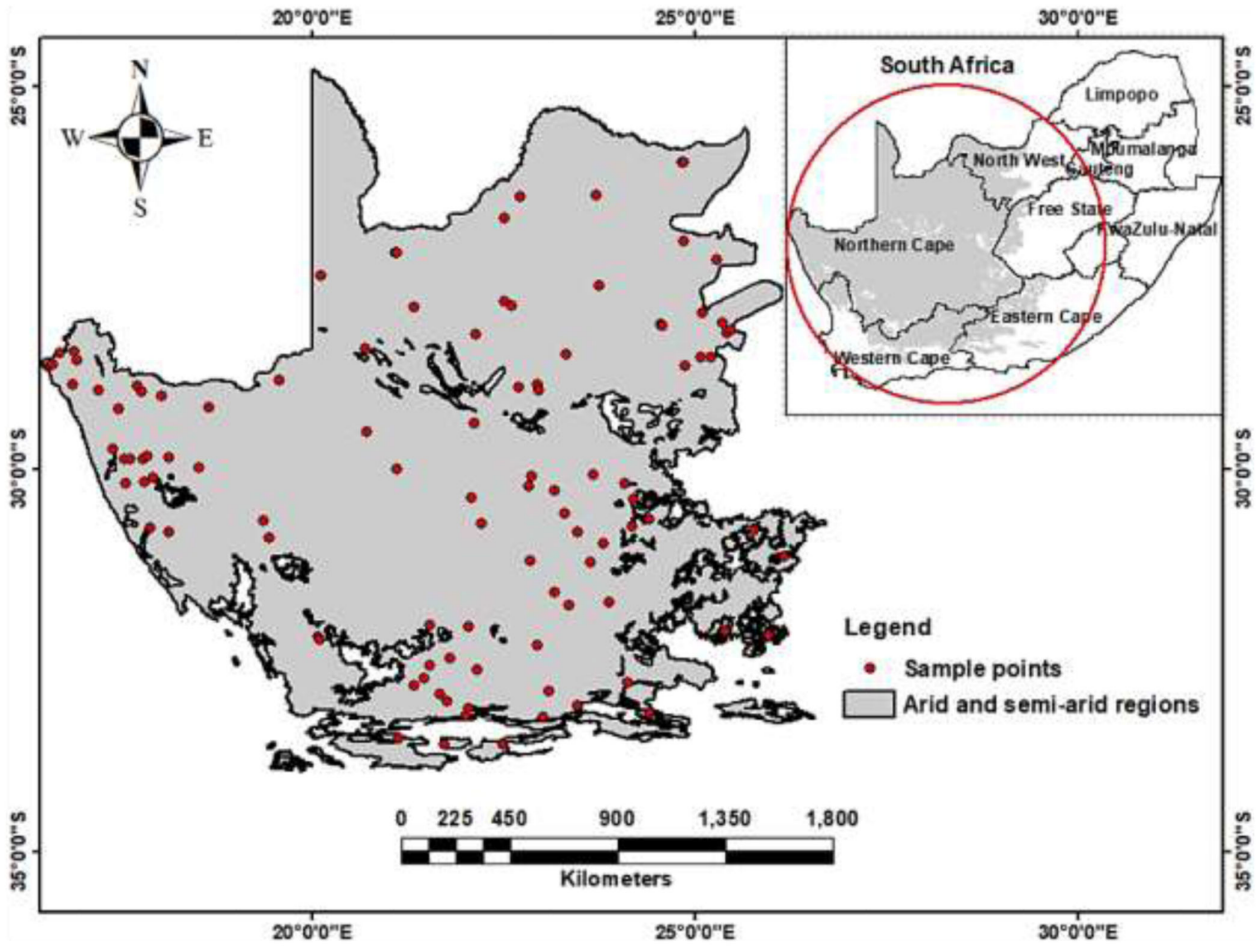
The arid and semi-arid region of South Africa and the spatial distribution of soil samples (red dots).

Arid and semi-arid landscapes in South Africa cover approximately 534,000 km^2^ and are predominately situated in the western part of the country (Figure 1) (Mucina and Rutherford, 2006). These landscapes have a semi-desert like climate characterized by relatively low annual rainfall (ranging from 70 to 500 mm) and high evaporation rates (Mucina and Rutherford, 2006). Consequently, water is limited, and soil quality is generally poor (Mucina and Rutherford, 2006). Additionally, the region experiences a mean annual temperature ranging between 20 and 26 °C (Richard *et al*., 2012; Desmet and Cowling, 1999). Moreover, the hot and dry conditions with low humidity and intense solar radiation lead to elevated rates of evapotranspiration that exacerbates water scarcity (Richard *et al*., 2012). As a result, the region is prone to frequent droughts that have severe impacts on local communities, agriculture, and biodiversity. Nevertheless, the study area encompasses a wide range of land uses, that include extensive livestock grazing, wildlife conservation, rain-fed cropping, as well as some areas of irrigated farming (Mucina and Rutherford, 2006). It is also home to large, protected areas like national parks and nature reserves (Mucina and Rutherford, 2006). The topography of the area is characterized by low-lying plains, rolling hills, and rocky outcrops, with several prominent mountain ranges, including the Cape Fold Belt (Nell and Van Huyssteen, 2014). Vegetation in the study area is diverse, ranging from open grasslands and savannas, to dense shrublands and thornveld (Rutherford, 2006). The dominant plant species include Acacia, Euphorbia, and Combretum, that are well-adapted to the harsh, arid and semi-arid conditions of the region (Mucina and Rutherford, 2006). The area also contains many unique and endemic plant species that include the succulent Karoo flora (Mucina and Rutherford, 2006). Overall, the arid and semi-arid region of South Africa is a complex and dynamic landscape shaped by a range of environmental and anthropogenic factors.

### 2.2 Soil data

An analysis of 242 soil profiles was conducted to examine the distribution of SOC content and bulk density across different soil depths (0-30cm, 30-60cm, 60-100cm, and 100-200cm) within South Africa’s arid and semi-arid regions. The soil profiles were sourced from the International Soil Reference Information Centre (ISRIC), an organization dedicated to providing comprehensive global information on soil properties, including SOC. The ISRIC soil database, which comprises of over 200,000 sample points from 173 countries, is regularly updated and incorporates various methods for determining SOC content, along with corresponding acquisition times and locations (Batjes *et al*., 2020). To ensure data consistency and standardization, ISRIC has established specific standardized procedures for inputting uniform soil profile data, which can be accessed through their website (https://www.isric.org/explore/wosis/accessing-wosis-derived-datasets). In this study, SOC stocks at various depths for each point were calculated using the formula (1), proposed by Pearson (2007):

(1) SOCaccumulation(t/h)=SOCconcemtrationxvolumedensityxsoildepth

### 2.3 Image data acquisition

#### 2.3.1 Sentinel 1 (SAR)

The study adopted the Sentinel-1 synthetic aperture radar (SAR) satellite data that forms part of the European Union’s Copernicus Programme launched on April 3, 2014. Sentinel-1 operates in the C-band frequency range and offers dual-polarization mode (“VV” and “VH”), enabling it to penetrate through clouds and vegetation (Yang and Guo, 2019; Shao *et al*., 2017). This characteristic makes it well-suited for mapping various soil properties, including SOC and terrain features. In this study, SAR data was preferred over optical data due to its ability to penetrate deeper into the soil, allowing for a more accurate assessment of SOC content at various depths. The satellite data has a 5 meters spatial resolution for the interferometric wide swath (IW) mode, covering a 250 km swath width (Mngadi *et al*., 2021). Meanwhile, the strip map (SM) mode has a 5 meters spatial resolution and an 80 km swath width, while the extra-wide swath (EW) mode has a 20 meters spatial resolution and a 400 km swath width (Mngadi *et al*., 2021; Ma *et al*., 2017).

The SAR images were pre-processed in Google Earth Engine (GEE) to generate high-quality images for both VV and VH polarizations. Median image collections of these polarizations (i.e., VV and VH) were aligned with the legacy soil profile data. To remove the effects of topography, terrain correction was applied to the images, thereafter, smoothing techniques were applied using the image-focal-median function to reduce radar data noise. Then, refined Lee speckle filtering was separately applied to the smoothed images for VV and VH polarizations to remove speckle noise. Composite images were created for both polarizations, and a Sentinel-1 Radar Vegetation Index (RVI) generated as an alternative to the Normalized Difference Vegetation Index (NDVI). The images were exported at a resolution of 20m for further analysis in R-studio (version 2023.03.1+446).

#### 2.3.2 Topo-climate metrics and soil physical properties

Previous studies (e.g., Odebiri et al., 2023b; Liu et al., 2021; Huang et al., 2019) have emphasized the influence of topographic and climatic factors on the dispersion of SOC. Essentially, these factors can be categorized as either local, non-local, or mixed variables (Li et al., 2018). Local variables evaluate the surface’s geometry, while non-local metrics establish the positions of points relative to another (Odebiri et al., 2023b). Topographic features that are both local and non-local are combined as mixed factors. In this study, eight significant terrain metrics (as outlined in Table 1), including Topographic Wetness Index (TWI), Direct insolation, Slope, General curvature, Catchment Area, Profile curvature, Aspect, and Elevation, were utilized based on previous studies in the area by Odebiri et al., (2020b) and Odebiri et al., (2023a) which identified them as key explanatory variables. These metrics were derived from an SRTM Digital Elevation Model (DEM) (Farr and Kobrick, 2000) using SAGA GIS (2.3.2) (Conrad et al., 2015) and ArcGIS Pro 2.8 software. Climate data (Table 1), such as average annual temperature and rainfall spanning over 30 years were obtained from WorldClim, available at http://www.worlclim.org. To match the spatial resolution of the Sentinel-1 data (20m), both the DEM and WorldClim datasets were resampled using the ‘raster resample’ function in ArcGIS Pro 2.8. Furthermore, physical soil characteristics known to be correlated with SOC stocks, such as soil type, coarse fragments, clay content, sand content, and silt content, were also considered as predictors in this study. These physical soil characteristics were extracted from the FAO soil portal (https://www.fao.org/soils-portal/) and ISRIC database (https://www.isric.org/) at four distinct soil sub-surface depths (0-30 cm, 30-60 cm, 60-100 cm, and 100-200 cm).

### 2.4 Analytical models

#### 2.4.1 Random Forest regression (RF)

The random forest (RF) algorithm is an ensemble machine learning technique that creates multiple decision trees and averages their results to predict a response variable (Breiman 2001). It builds decision trees using a random subset of the training data through bootstrapping, creating diverse trees with different rules and predictions (Odebiri *et al*., 2020a). At each node of the tree, a random subset of features is considered for splitting. Once the decision trees are created, the final prediction is made by averaging the predictions of all the trees (Odindi et al., 2016). RF is suitable for handling large datasets with high-dimensional features, missing data, and outliers (Emadi *et al*., 2020). It can also determine the importance of features and prevent overfitting (Forkuor et al 2017). The key parameters to optimize in RF are *ntree* (the number of regression trees built using a bootstrap sample of the observations, and has a default value of 500); *mtry* (the number of different predictors tried at each node, and is calculated as the total number of variables divided by three); and the *nodesize* (which represents the minimum size of the regression trees’ terminal nodes, and has a default value of one) (Ließ et al., 2016; Mutanga et al., 2012). The value range of RF hyper-parameter tuning used in this study is shown in Table 2.

#### 2.4.2 Extreme Gradient Boosting (XGBoost)

Extreme Gradient Boosting (XGBoost) is a popular ensemble learning algorithm that has been widely used in various machine learning tasks, including regression. It works by iteratively adding weak learners to a base model to improve its performance (Emadi *et al*., 2020). A weak learner is a simple model that has a high bias and low variance (Chen *et al*., 2016). In XGBoost, the weak learners are decision trees. The algorithm first fits a decision tree to the training data and then calculates the errors between the predicted and actual values (Fan *et al*., 2018). It then adds a new decision tree to the model that focuses on reducing the errors made by the previous tree (Emadi *et al*., 2020). This process is repeated until the desired number of trees is reached, or until the model’s performance stops improving. XGBoost has several hyper-parameters that can be tuned to improve the model’s performance. These hyper-parameters include the number of trees, learning rate, maximum depth of each tree, subsample ratio, and column subsample ratio (Fan *et al*., 2018). The number of trees determines the number of weak learners added to the model, while the learning rate controls how much each tree contributes to the overall model (Emadi *et al*., 2020). The maximum depth of each tree limits the number of splits the tree can make, and the subsample ratio controls the fraction of training data used to fit each tree. The column subsample ratio controls the fraction of columns used to fit each tree. XGBoost is known for its accuracy, efficiency in handling large datasets, robustness to outliers, and ability to handle missing data (Chen *et al*., 2016). The specific XGBoost hyper-parameters used in this study and their corresponding ranges are presented in Table 2.

### 2.5 Model evaluation metrics

In this study, the total dataset (N = 242) was separated into 70% (n = 169) for calibration model and 30% (n = 73) validation model. Furthermore, two accuracy metrics were used to assess the performance and generalizability of the models. The Root Mean Square Error (RMSE) was used to determine the average magnitude of errors between the predicted and actual values, while the Coefficient of Determination (R^2^) was used to determine the proportion of variance in the actual values that were accounted for by the model. A model with a high R^2^ value and a low RMSE value is the best fit. The mathematical expressions for these metrics can be found in Odebiri *et al*., (2022b). Furthermore, the contribution of each covariate to the variability of SOC stocks at each depth was evaluated to determine their importance.

## 3 Results

### 3.1 Descriptive statistics of the SOC data

An overview of the descriptive statistics of the soil profile data (242 samples) used in this study at the four depths (0-30 cm, 30-60 cm, 60-100 cm, and 100-200 cm) are presented in Table 3. Notably, the variance of SOC stocks was low in the topsoil (0-30 cm), accounting for approximately 30% of the total variance. In contrast, the remaining depths exhibited considerably higher variances (> 60%). The SOC data across all depths exhibited high levels of skewness and kurtosis, indicating a departure from a normal distribution. To address this issue, a natural logarithm transformation (Log10) was applied to improve the data’s normality, resulting in new values for skewness and kurtosis that reflected a more symmetric distribution. After the predictive analysis, the transformed SOC data was then reverse transformed to restore it to its original scale. The reverse-transformation was necessary to ensure the interpretability of the results and to facilitate meaningful comparisons with the original data. The results of the study reveal variations in SOC content across different depths. In the topsoil layer (0-30 cm), SOC ranged from 8 to 45 t/ha, with an average value of 19.37 t/ha (Table 3). Deeper into the soil profile, the 30-60 cm depth exhibited SOC values ranging from 6 to 86 t/ha, with an average of 19.72 t/ha (Table 3). In the 60-100 cm depth range, SOC values ranged from 7 to 109 t/ha, with an average of 25.56 t/ha (Table 3). Finally, the deepest layer (100-200 cm) showed the widest range of SOC values, with SOC ranging from 18 to 318 t/ha, with an average of 77.76 t/ha (Table 3). These findings demonstrate the heterogeneity of SOC distribution within the soil profile, with higher levels of SOC observed at greater depths.

### 3.2 Evaluation and model performance

**Figure 2:**
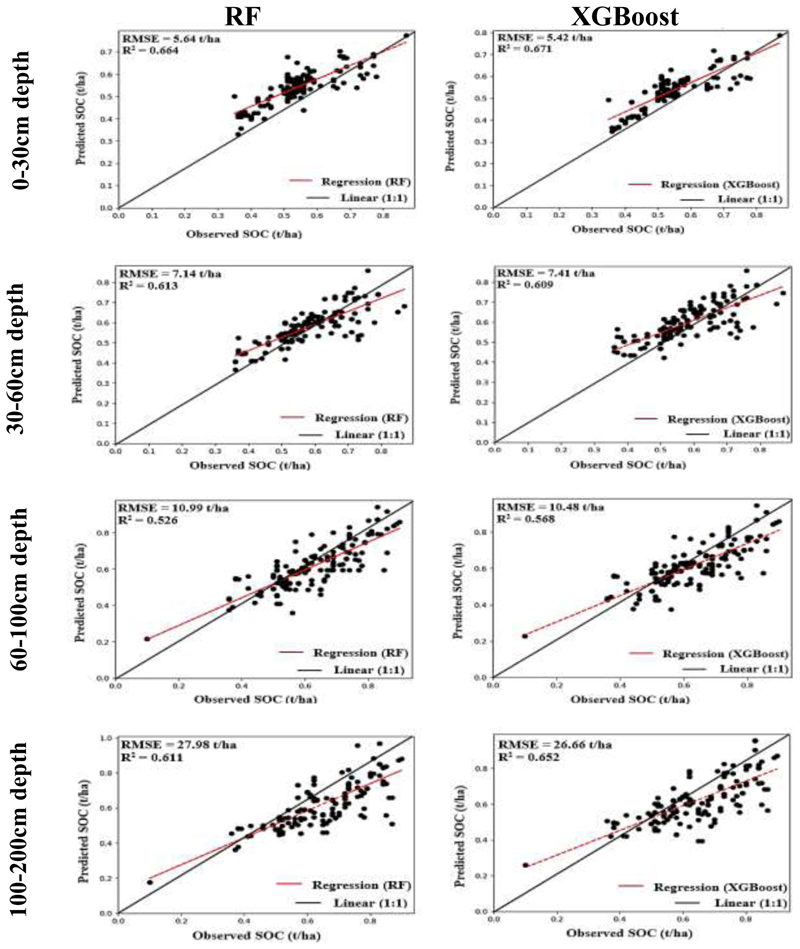
Scatter plots of the training data showing observed Soil Organic Carbon (SOC) against predicted SOC, obtained from Random Forest (RF) and Extreme gradient boosting (XGBoost) models, within soil depths of 0-30 cm, 30-60 cm, 60-100 cm, and 100-200 cm.

The performance of two different models, Random Forest (RF) and Extreme Gradient Boosting (XGBoost), in estimating SOC stocks at various depths is presented in Table 4 and Figure 2. Generally, XGBoost consistently outperformed RF in terms of accuracy and goodness-of-fit. Specifically, for the 0-30cm depth range, the XGBoost model outperformed the RF model, demonstrating a lower RMSE of 7.12 t/ha and higher R^2^ values of 0.671 for the train data and 0.617 for the test data. In contrast, the RF model achieved an RMSE of 7.36 t/ha for the test data, with corresponding R^2^ values of 0.664 for the train data and 0.603 for the test data (Table 4 and Figure 2). Similarly, for the 30-60cm depth range, XGBoost had a better RMSE (7.41 t/ha) and R^2^ value (0.580) for the test data compared to the RF model (Table 4). In comparison, the RF model obtained an RMSE of 7.89 t/ha, with a corresponding R^2^ value of 0.578 for the test data at a 30-60cm soil depth (Figure 2). Thereafter, for the 60-100cm depth range, the XGBoost model demonstrated superior performance compared to the RF model. It achieved a lower RMSE of 10.48 t/ha and higher R^2^ values of 0.568 for the train data and 0.543 for the test data (Table 4). Conversely, for the same depth range, the RF model obtained an RMSE of 10.99 t/ha for the train data and 11.23 t/ha for the test data, with corresponding R^2^ values of 0.526 and 0.501, respectively. Finally, for the deepest soil depth range of 100-200cm, XGBoost exhibited a lower RMSE (26.66 t/ha) and higher R^2^ values (0.652 for train data, 0.601 for test data) compared to the RF model. Overall, both RF and XGBoost models showed reasonable performance in estimating SOC stocks at different depths. However, XGBoost consistently exhibited better accuracy and goodness-of-fit, as indicated by lower RMSE values and higher R^2^ values. These findings suggest that the XGBoost model may be more suitable for reliably predicting SOC stocks at various depths in this study.

### 3.3 Evaluation of variable importance at various soil depths

**Figure 3:**
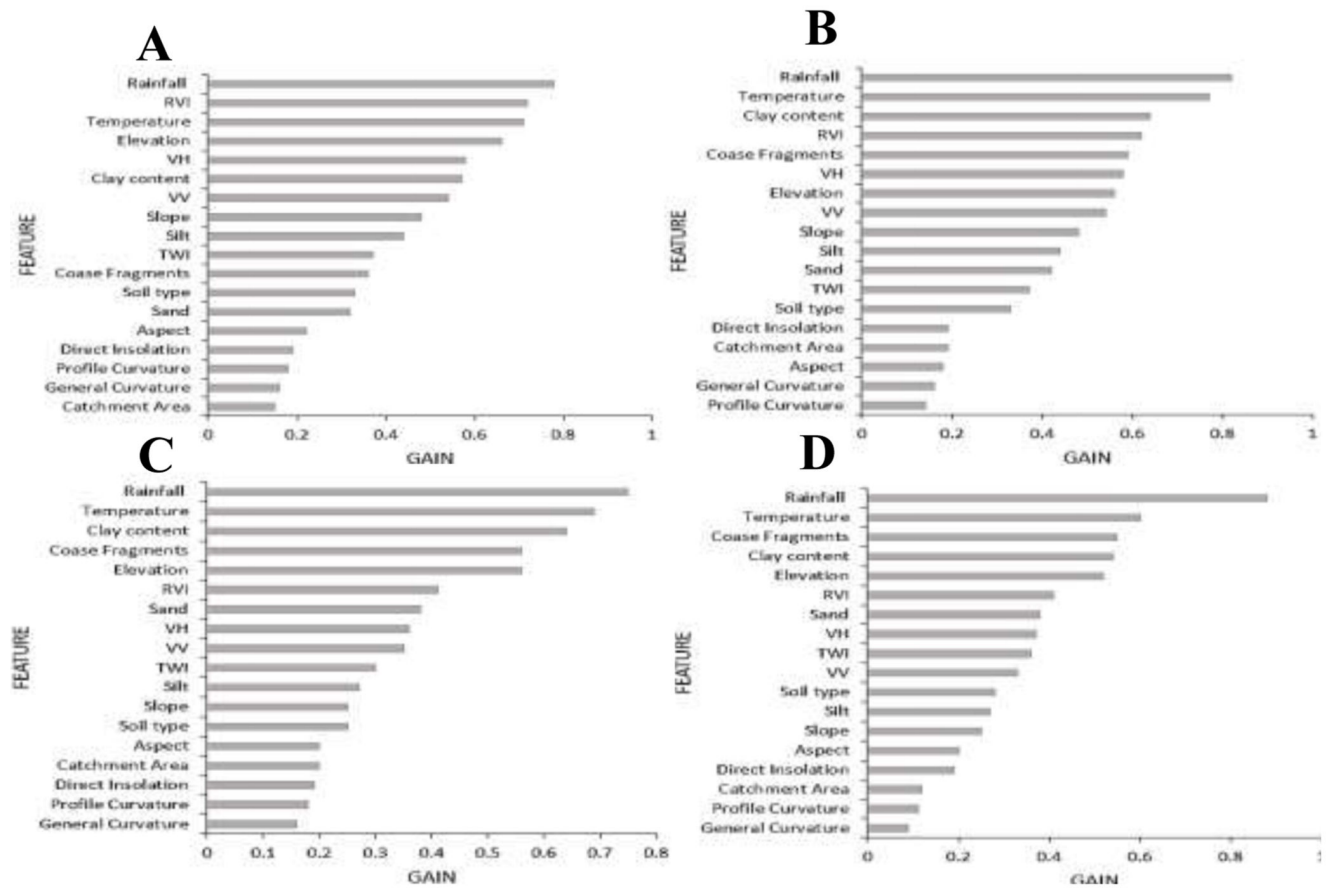
XGBoost model variable importance ranking for SOC stocks distribution across four depths with A, B, C and D representing soil depths of 0-30cm, 30-60cm, 60-100cm, and 100-200cm, respectively.

The importance of each covariate in estimating SOC at different depths was obtained from the best performing model (XGBoost) and is depicted in Figure 3. The most important variables for the topsoil (0-30cm) were rainfall, RVI, temperature, elevation, and VH (Figure 3). However, as the depth increased, there was a noticeable shift in the most influential variables, indicating that the drivers of SOC stocks differ between the topsoil and deeper soil layers in the study area. Consequently, for the second depth (30-60cm), rainfall remained the most important variable, followed by temperature, clay content, RVI, and coarse fragments (Figure 3). At the third depth (60-100cm), the most significant variables were rainfall, temperature, clay content, coarse fragments, and elevation (Figure 3). In the deepest range (100-200cm), rainfall and temperature were once again the two most important variables, with coarse fragments, clay content, and elevation also playing crucial roles in the amount of SOC. Other variables such as silt, VV, TWI, sand, soil type, and slope exhibited significant contributions to the model across all depths, although they did not rank among the top five variables. These findings indicate that the most important variables influencing SOC stocks vary depending on soil depth. Moreover, climatic variables, particularly rainfall and temperature, consistently emerged as the primary drivers of SOC stocks in the study area. These results underscore the importance of considering soil depth when developing models for predicting SOC stocks.

### 3.4 SOC storage potential and distribution at different soil depths

Table 5 displays the distribution of SOC stocks at different soil depths, including the percentage of the total SOC stocks and the corresponding minimum, mean, and maximum values. Generally, the results highlight the diversity in SOC stocks within arid and semi-arid regions, as evidenced by the variations in SOC distribution across the different depth ranges. Specifically, for the 0-30 cm depth range, SOC stocks account for 15.98% of the total, with a minimum value of 6.24 t/ha, a maximum value of 44.97 t/ha, and an average value of 18.98 t/ha (Table 5). At the 30-60 cm depth range, the percentage of total SOC stocks increases to 19.33%, with a minimum value of 5.58 t/ha, a maximum value of 89.98 t/ha, and a mean value of 19.14 t/ha (Table 5). At the 60-100 cm depth range, the percentage of total SOC stocks further rises to 23.68%, with a minimum value of 7.25 t/ha, a maximum value of 103.64 t/ha, and an average value of 28.44 t/ha (Table 5). Finally, the deepest range of 100-200 cm exhibits the highest percentage of total SOC stocks at 41.01%, with a minimum value of 15.57 t/ha, a maximum value of 299.21 t/ha, and a mean value of 64.30 t/ha (Table 5). These findings demonstrate that the distribution of SOC stocks varies across different depth ranges. SOC stocks generally increase with depth, with the deepest layer (100-200 cm) containing the highest proportion of SOC stocks. The minimum, mean, and maximum values of SOC stocks also exhibit significant variations at the different depth ranges, highlighting the heterogeneity of SOC distribution within the soil profile of arid and semi-arid environments.

**Figure 4:**
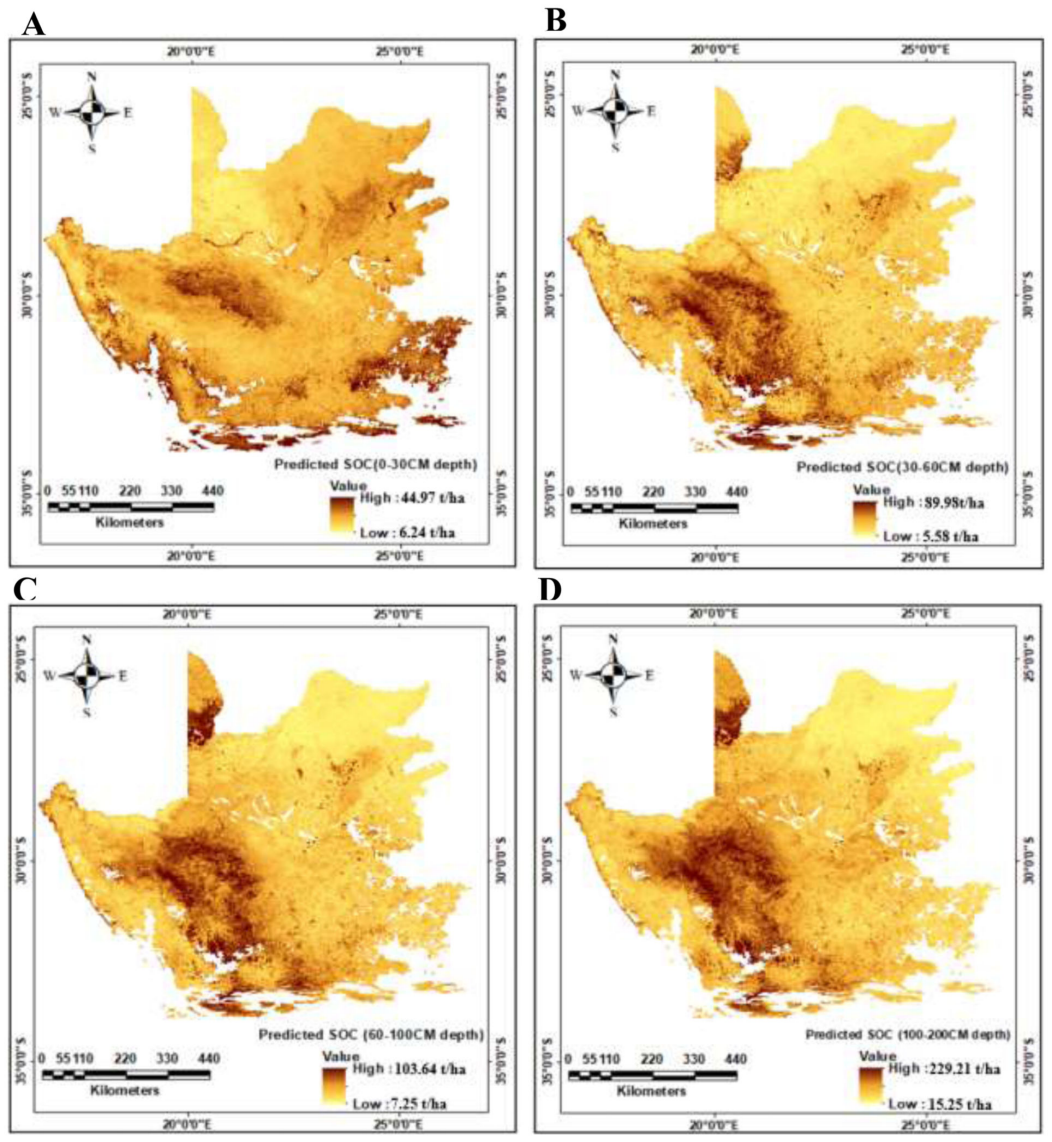
The spatial and sub surface distribution of soil organic carbon (SOC) stocks across different soil depths within the arid and semi-arid areas of South Africa. A, B, C and D represents different soil depths of 0-30cm, 30-60cm, 60-100cm, and 100-200cm respectively expressed in tonnes per hectare (t/ha).

The spatial distribution of SOC stocks within arid and semi-arid regions across each of the different depth ranges is depicted in Figure 4. The figures display a prominent concentration of darker colours in the central and northernmost regions, indicating an overall increase in SOC stocks with depth. However, a contrasting pattern is observed along the north-eastern axis, where SOC stocks decrease as depth increases. These findings suggest a consolidation of SOC stocks in the central interior of the Northern Cape, as well as the northern parts of the Western Cape province as soil depth increases.

## 4 Discussion

The susceptibility of South Africa’s arid and semi-arid regions to increased desertification and soil degradation emphasizes the need for accurate regional SOC data to improve land-use management frameworks and enhance climate change mitigation efforts (Mbow et al., 2017). Anthropogenic and environmental factors, including climate change, topography, soil characteristics, and land-use modifications, have been shown to not only accelerate desertification and soil degradation within these landscapes, but also affect SOC (Cerri et al., 2007, Rosinger et al., 2023, Shoba and Ramakrishnan, 2016). Consequently, evaluating SOC stocks and their response to these factors is crucial amid rapid climate and land-use changes (Lorenz and Lal, 2005, Poeplau et al., 2011). Accurate SOC assessments, however, requires examining different soil depths due to SOC’s uneven distribution within the soil profile (Luo et al., 2021). This knowledge is vital for developing effective land management strategies to combat climate change and promote sustainable soil and land use practices. To derive precise SOC stock estimates in South Africa’s arid and semi-arid regions, this study utilized an ensemble of decision-tree algorithms, integrating multiple covariates such as Sentinel 1 remotely sensed data, topo-climatic conditions, and soil physical properties.

### 4.1 The distribution of SOC stocks at different depths in South Africa’s Arid and Semi-Arid landscapes

The results of this study showed that significant amounts of SOC are distributed across various soil depths in South Africa’s arid and semi-arid regions. Specifically, 15.98%, 19.33%, 23.68%, and 41.01% of SOC are stored at the 0–30 cm, 30–60 cm, 60–100 cm, and 100–200 cm soil depths, respectively (Table 3). The subsoil and the substratum layers were found to contribute significantly to total SOC stocks within the region, accounting for more than 80% of the total SOC stocks, with half of this stored within the deep substratum layer (100-200 cm). These findings demonstrate the distinct distribution patterns of SOC within arid and semi-arid environments of South Africa and correspond to similar findings by Liu et al. (2017) and Amirian Chakan et al. (2017), who observed higher SOC concentrations at greater soil depths in arid environments. This outcome could be attributed to the unique biological and environmental factors in South Africa’s arid and semi-arid regions like the Northen Cape, known for its dry climate, limited rainfall, and sparse vegetation (Hua et al., 2019), which collectively have the potential to influence SOC dynamics (Jiang et al., 2017, Ardö and Olsson, 2003). For instance, in the upper soil layers (0-30 cm), where evaporation rates are high, the elevated temperatures akin to these environments accelerate organic matter decomposition, resulting in lower SOC content (Albaladejo et al., 2013). However, as you move deeper into the soil profile (30-200 cm), temperature fluctuations become less severe, and moisture availability increases due to greater water infiltration (Larsson and Jarvis, 1999). These conditions slow down decomposition rates and preserve organic matter, leading to higher SOC at greater depths. This corresponds to a study in arid Iran by Amirian Chakan et al. (2017), who observed increased SOC in deeper soil layers (beyond 60 cm) due to reduced decomposition rates, limited oxygen, and moisture fluctuations.

Similarly, the soil types and texture, including the proportions of sand, silt, and clay particles, significantly influence SOC dynamics within arid and semi-arid regions (McAuliffe et al., 2018). For instance, Soils rich in clay and humus, such as Vertisols, have higher SOC content and sequestration capacity (Wiesmeier et al., 2015, Kögel-Knabner and Amelung, 2021), while soils with higher clay and silt content, offer increased water-holding capacity and a larger surface area for organic matter adsorption and stabilization (Wiesmeier et al., 2019). This promotes the absorption, retention, and accumulation of organic matter, enhancing SOC stocks (Sarkar et al., 2018). A study by Liu et al. (2017), which examined SOC distribution across different soil depths in China, also found that SOC content increased significantly with soil depth. This increase was linked to the greater accumulation of fine particles and clay minerals, which enhance organic matter retention. Conversely, sandy soils, like Entisols, have lower water-holding capacity and less SOC, with this impact more noticeable in shallower layers (0-30 cm) as opposed to deeper soil layers (Yost and Hartemink, 2019). Moreover, soils rich in minerals like iron and aluminium oxides, such as Ultisols, have higher cation exchange capacity and improved soil aggregation, which protect and stabilize organic matter (Sarkar et al., 2018), leading to increased SOC, especially deeper into the soil (Kögel-Knabner and Amelung, 2021). Furthermore, topographic factors like slope and aspect may influence SOC distribution in arid and semi-arid environments especially in the topsoil (Liu et al., 2011). Specifically, regions with gentle slopes promote improved water infiltration and reduced surface runoff (Schwanghart and Jarmer, 2011), while aspect influences solar radiation exposure and moisture availability (Jakšić et al., 2021, Xue et al., 2018). Collectively, these factors facilitate the transport of organic matter deeper into the soil profile, resulting in higher SOC content at greater depths. Additionally, South Africa’s semi-arid regions are dominated by shrublands with some areas like the karoo region in the eastern cape containing Spekboom (*Portulacaria afra*) (Mucina and Rutherford, 2006). Known for its ability to thrive in harsh climates, rapid growth, and its proclivity for substantial litter production (Mills et al., 2015), this plant species has the capacity to sequester approximately 168 metric tons of carbon per hectare (Mills and Fey, 2004). Overall, a combination of these factors contributes to the complex sub-surface accumulation and distribution patterns of SOC in South Africa’s semi-arid and arid landscapes, where the vegetation composition and unique environmental conditions influence the dynamics of organic matter inputs, decomposition rates, and translocation.

The spatial distribution of SOC stocks within South Africa’s arid and semi-arid regions was also examined. The models revealed that concentration and isolation of SOC stocks in the central regions of the Northern Cape province increases with soil depth. This distinctive pattern can be attributed to various *in situ* factors and is shaped by the region’s particular climate, environment, and soil texture (Carr et al., 2013, Dzikiti et al., 2013). Specifically, parts of the Northern Karoo, characterized by higher elevations (Botes et al., 2006), could potentially receive increased precipitation, creating conditions within the topsoil conducive to SOC sequestration, in turn supporting higher biomass productivity and greater organic matter input (Minasny et al., 2017) within Karoo shrublands. Furthermore, the Northern Cape region encompasses various soil types that include inceptisols, aridisols and xerosols (Moore et al., 1985). Inceptisols can facilitate deep SOC storage through B horizon development and illuviation, while Aridisols and Xerosols accumulate organic matter at different soil depths via processes like colluviation and windblown sediment deposition, which are influenced by the specific conditions unique to arid and semi-arid regions (Moore et al., 1985). In contrast, other areas along the north-eastern axis may have different soil types or textures that are less conducive to SOC accumulation, resulting in lower SOC stocks with increasing depth. Overall, the combination of arid and semi-arid climatic conditions, levels of elevation and precipitation, and distinct soil properties within the central parts of the Northern Cape contribute to the regions SOC dynamics. These findings highlight the importance of considering the local environmental characteristics when assessing SOC distribution.

Nevertheless, land-use changes in South Africa’s arid and semi-arid areas can accelerate desertification and soil degradation, profoundly affecting SOC dynamics and stocks (Albaladejo et al., 2013). The conversion of natural vegetation to croplands, overgrazing, or urbanization can disrupt SOC accumulation and decomposition, resulting in reduced SOC stocks and compromised soil health (Lal, 2015). For example, conversion to cropland disrupts soil through increased disturbance, plant removal, and erosion, resulting in substantial SOC losses from faster decomposition and reduced plant residue input (Lal, 2015). Meanwhile, intensive tillage and chemical fertilizers also impact SOC storage potential in these areas (Powlson et al., 2012). Overgrazing further contributes to SOC losses by reducing vegetation cover, compacting soil, and increasing erosion (Mchunu and Chaplot, 2012). Lastly, urbanization removes vegetation cover and seals the soil, resulting in topsoil loss and disrupted moisture regimes, affecting microbial activity and organic matter decomposition (Ferreira et al., 2022). Thus, anthropogenic activities and land-use change can significantly impact SOC stocks and alter ecosystem functioning (Leifeld et al., 2005). However, understanding these impacts and implementing conservation agriculture practices, like reduced tillage and cover crops, as well as promoting sustainable grazing practices can enhance SOC storage and mitigate land-use change effects (Lal, 2004, Plaza-Bonilla et al., 2015). This is crucial for preserving SOC stocks, maintaining soil health, and addressing climate change in these vulnerable landscapes.

### 4.2 Model Performance and Variable Importance

The study found a general decline in model accuracy with increasing soil depth for both the Extreme Gradient Boosting (XGBoost) and Random Forest (RF) models. In the XGBoost SOC models, RMSE increased from 5.42t/ha at 0-30cm to 26.66t/ha at 100-200cm, while the RF model followed a similar trend, with RMSE increasing from 5.64t/ha at 0-30cm to over 27t/ha at 100-200cm (Figure 2). This outcome indicates a weakening relationship between environmental factors and SOC stocks as soil depth increases, which aligns with earlier studies by (Nussbaum et al., 2014, Li et al., 2023, Taghizadeh-Mehrjardi et al., 2016). This may be attributed to variations in soil properties (e.g., soil texture, structure, moisture) at different depths, weakening the correlation between environmental factors and SOC stocks (Liang et al., 1996, Coblinski et al., 2020). This is corroborated by the higher variability in SOC stocks observed at lower depths (Table 3). Additionally, soil formation processes and biological factors become more influential as soil depth increases (Van Breemen and Buurman, 2002, Cornelis and Delvaux, 2016), contributing towards greater soil heterogeneity (Reynolds et al., 1997), which in turn result in a wider range of SOC stocks (as seen in Table 3). These factors make precise predictions more challenging and introduce measurement errors, thereby reducing model accuracy.

Nevertheless, the results showed that XGBoost consistently outperformed RF in terms of accuracy and goodness-of-fit at all soil depths, as indicated by lower RMSE values and higher R^2^ values (Figure 2). This was unsurprising, as XGBoost is known for its ability to capture complex nonlinear relationships between input and the target variables (Emadi *et al*., 2020). As previously outlined in section 4.1, SOC dynamics are influenced by various interacting factors, such as soil properties, climate, and vegetation, which can exhibit nonlinear patterns. Subsequently, XGBoost’s flexibility in modelling nonlinear relationships allowed it to better capture the intricate relationships between input features and SOC content at different soil depths (Emadi *et al*., 2020). Furthermore, XGBoost is based on the gradient boosting framework, which involves sequentially adding weak learners (decision trees) to the ensemble, each focusing on correcting the errors made by the previous learners (Chen *et al*., 2016). This iterative process enables XGBoost to learn from the mistakes and adjust its predictions accordingly (Fan *et al*., 2018). This approach can potentially improve the accuracy of SOC estimation, especially when there are complex relationships and interactions among predictors. Moreover, XGBoost implements techniques such as L1 and L2 regularization and tree pruning (Chen *et al*., 2016), which prevent overfitting and reduce the model’s sensitivity to noise and outliers in the data by effectively managing the model’s overall complexity (Chen *et al*., 2016).

To accurately estimate SOC stocks across different depths, it’s crucial to consider various factors like climate, topography, and soil characteristics (Tayebi et al., 2021, Taghizadeh-Mehrjardi et al., 2016). While climatic, topographic, soil type, and texture variables were all essential predictors of SOC, their relative influence varied with soil depth. Despite this, climatic variables, particularly rainfall and temperature, consistently emerged as the most important predictors of SOC across all depths. This highlights the significant role of climate in influencing SOC stock variability in these environments — as discussed in section 4.1. Nevertheless, remotely sensed variables such as the Ratio Vegetation Index (RVI), Vertical-Horizontal polarization (VH), Vertical-Vertical (VV) polarization, and Terrain Wetness Index (TWI) were also found to be important for estimating SOC within arid and semi-arid areas. In arid and semi-arid areas, where vegetation cover is often sparse, the RVI can be used to estimate aboveground biomass and vegetation productivity (Li et al., 2016). This is closely related to organic inputs into the soil, which in turn influences the amount of SOC within these regions. A recent study by Matinfar et al. (2021) also found RVI to be an important predictor of SOC. However, in our study, the relevance of RVI decreased with depth since vegetation-related signals tend to attenuate and become less significant below the soil surface (Moran et al., 2000). VH and VV polarization, which refer to the polarizations of Synthetic Aperture Radar (SAR) data, can penetrate through vegetation and provide information on soil moisture, roughness, and surface scattering characteristics (Kornelsen and Coulibaly, 2013, Srivastava et al., 2006). In arid and semi-arid regions, soil moisture is a crucial factor affecting organic matter decomposition rates and SOC dynamics (Gorooei et al., 2023). Subsequently, VH and VV polarizations can be used to determine soil moisture content (Ezzahar et al., 2019), which indirectly relates to SOC content. However, the influence of SAR data on SOC estimation may diminish with increasing soil depth as the signal weakens and interacts with more layers of the soil profile (Kumar et al., 2019). Terrain Wetness Index (TWI) is a topographic index that characterizes the wetness or moisture availability within a specific terrain (Schmidt and Persson, 2003). It considers factors such as slope, aspect, and flow accumulation. In arid and semi-arid areas, where moisture availability strongly influences vegetation growth and organic matter inputs (Prasad et al., 2016), TWI was found to provide insights into areas with higher moisture accumulation potential. This high moisture availability is associated with increased organic matter decomposition rates and subsequent SOC accumulation (Wong et al., 2010). However, the relevance of TWI was found to vary with depth as the influence of topography on soil moisture availability differs at different soil depths.

It is important to note that the relevance and importance of these remote sensing variables may vary at different soil depths due to a decrease in remote sensing signals as they penetrate deeper into the soil profile. Consequently, signals related to aboveground vegetation, such as RVI, may become less significant or undetectable at greater soil depths. Moreover, as remote sensing signals interact with multiple soil layers, their relationship with SOC can change. In this instance, factors such as soil texture, mineral composition, and water content can alter the signal response and its relation to SOC content. As a result, the relationships between remote sensing variables and SOC were found to vary significantly across different soil depths (Figure 3). Thus, to accurately estimate SOC at different points within the soil profile and adequately capture the complexities of SOC dynamics in arid and semi-arid regions, we found it essential to integrate remote sensing datasets with topo-climatic features, *in-situ* soil sampling and depth-specific analysis.

Nevertheless, we acknowledge that there are some uncertainties in the SOC stock models due to the inherent variability that may exist within the legacy data that was used (Owusu et al., 2020). Legacy soil data, although useful for digital soil mapping, poses challenges due to its uneven distribution and age, resulting in wide prediction intervals (Owusu et al., 2020). The uneven distribution of data at country scale can also affect the accuracy of the predicted variables. Consequently, future studies should focus on improving sampling schemes, employing more advanced machine learning and remote sensing techniques, and integrating optical and radar data to enhance the accuracy of SOC estimation. Lastly, comprehending the complex interactions among climate, topography, soil properties, and remote sensing variables is crucial for developing precise models of SOC stocks and fluxes in arid and semi-arid environments.

## 5 Conclusion

This study highlighted the importance of quantifying and understanding SOC stocks in arid and semi-arid regions of South Africa for effective climate change mitigation. The vulnerability of these regions to desertification and soil degradation necessitates accurate assessments of SOC concentrations and their distribution at various soil depths. The study reveals distinct distribution patterns of SOC within the arid and semi-arid environments of South Africa. Specifically, SOC stocks were found to increase with soil depth, with significant amounts stored in the subsoil and deep substratum layers. The unique conditions of arid and semi-arid regions, including its dry climate and sparse vegetation, contribute to the accumulation of SOC at greater depths, while factors such as climate, soil texture, soil types, mineral composition, and topography were found to significantly influence SOC dynamics and storage in these regions. The spatial distribution of SOC stocks within arid and semi-arid regions also vary. The central parts of the Northern Cape province showed higher concentrations of SOC. The study also assessed the performance of two machine learning models, specifically Extreme Gradient Boosting (XGBoost) and Random Forest (RF), in estimating SOC stocks at different soil depths. XGBoost was found to consistently outperform RF, demonstrating its ability to capture complex nonlinear relationships between input variables and SOC content. However, the model’s accuracy declined with increasing soil depth due to variations in soil properties and other environmental factors. Moreover, the study identified several key variables that influenced SOC estimation at different soil depths. Specifically, climatic variables (i.e., rainfall and temperature) emerged as the primary drivers of SOC stocks throughout the soil profile. Other factors such as soil texture, elevation, coarse fragments, and remote sensing variables also played significant roles in SOC stocks. Overall, this study provides valuable insights into the distribution of SOC stocks and the factors influencing SOC dynamics in arid and semi-arid regions of South Africa. Understanding these patterns are crucial for developing effective land management strategies, preserving SOC stocks and maintaining soil health in these vulnerable regions.

## Figures and Tables

**Table 1:** Predictors covariates and their description/formula

Covariates	Description/formula	Reference
		
**Sentinel 1 SAR data**		
VV Polarization	Ascending IW swath mode, 20m resolution	ESA
VH Polarization	Ascending IW swath mode, 20m resolution	ESA
Radar Vegetation Index (RVI)	4*VH/(VV+VH)	Kumar *et al.,* (2013)
		
**Topo-climate**		
Elevation (DEM)	Ground height (m)	Davy and Koen (2014)
Slope	The steepness of the ground	Li *et al.,* (2014)
Aspect	Slope cardinal direction	Rezaei and Gilkes, (2005)
Topographic wetness index (TWI)	Steady state wetness index	Lang *et al.,* (2013)
General curvature (Gen Curv)	Curvature both horizontally and vertically	Li *et al.,* (2014)
Direct Insolation (Dir Ins)	Potential Incoming insolation	Rodriguez *et al.,* (2002)
Profile curvature (Pro Curv)	Vertical rate of change of slope	Ritchie *et al.,* (2007)
Catchment Area	Water runoff velocity and volume	Kasai *et al.,* (2001)
Rainfall	Mean annual precipitation	Odebiri *et al.,* (2020b)
Temperature	Mean annual temperature	Odebiri *et al.,* (2020b)
		
**Soil physical properties**		
Coarse fragments	Primary soil particle larger than 2 mm in nominal diameter	Hengl *et al.,* (2017)
Clay content	Mineral particles smaller than 2 µm	Hengl *et al.,* (2017)
Sand content	Natural granular material made up of finely divided rock and mineral particles	Hengl *et al.,* (2017)
Silt content	Very small particles left as water sediment	Hengl *et al.,* (2017)
Soil type	Soil classification based on the percentage of sand, silt, and clay in its composition	FAO soil data portal

**Table 2:** Hyper-parameters for both Random Forest and Xtreme Gradient Boosting used.

Algorithm	Hyper-parameters	Parameter as used	Parameter Description
RF	*Mtry*	1–20	Input variables number
	*Ntree*	100–1000	Number of trees
	*Node size*	1	Size for the regression trees terminal nodes
XGBoost	*Booster*	Gbtree	The model type
	*Maxdepth*	3–10	The tree’s depth
	*Min_child_weight*	0–5	The smallest sum of all observational weights
	*Colsample_bytree*	0.5–1	The quantity of variables a tree is given
	*Subsample*	0.5–1	The quantity of samples given to a tree
	*Eta*	0.01–0.3	Learning rate
	*Gain*	–	Variable Importance Measure

**Table 3:** SOC stock data summary statistics (t/ha); new skewness and kurtosis for the log transformed in brackets.

SOC depths(cm)	Min	Max	Mean	Standard deviation	Coefficient of variation (%)	Kurtosis	Skewness
0-30	8	45	19.37	5.78	29.84	2.22 (0.23)	0.85 (0.15)
30-60	6	86	19.72	11.91	60.40	10.81(0.38)	2.67(0.63)
60-100	7	109	25.56	16.20	63.38	7.70(0.27)	2.20(0.22)
100-200	18	318	77.76	48.27	62.08	6.58(0.21)	2.04(0.14)

**Table 4:** Summary of the SOC stocks RF and XGBoost models at four different soil depths (0-30cm, 30-60cm, 60-100cm, and 100-200cm), for both the train and test data.

SOC depths(t/ha)	Random Forest (RF)				Extreme Gradient Boosting (XGBoost)			
	Train data		Test data		Train data		Test data	
	RMSE	R^2^	RMSE	R^2^	RMSE	R^2^	RMSE	R^2^
0-30cm	5.64	0.664	7.36	0.603	5.42	0.671	7.12	0.617
30-60cm	7.14	0.613	7.89	0.578	7.41	0.609	7.69	0.580
60-100cm	10.99	0.526	11.23	0.501	10.48	0.568	10.74	0.543
100-200cm	27.98	0.611	31.10	0.579	26.66	0.652	29.55	0.601

**Table 5:** Summary of estimated soil organic carbon (SOC) content and stocks across four soil depths (0-30 cm, 30-60 cm, 60-100 cm, and 100-200 cm).

SOC depths(cm)	Total SOC (%)	Min (t/ha)	Mean(t/ha)	Max(t/ha)
0-30	15.98	6.24	18.98	44.97
30-60	19.33	5.58	19.14	89.98
60-100	23.68	7.25	28.44	103.64
100-200	41.01	15.57	64.30	299.21

## Data Availability

Data are available from the authors upon request.
